# CT texture features are associated with overall survival in pancreatic ductal adenocarcinoma **–** a quantitative analysis

**DOI:** 10.1186/s12880-017-0209-5

**Published:** 2017-06-19

**Authors:** Armin Eilaghi, Sameer Baig, Yucheng Zhang, Junjie Zhang, Paul Karanicolas, Steven Gallinger, Farzad Khalvati, Masoom A. Haider

**Affiliations:** 1 Department of Medical Imaging and Sunnybrook Research Institute, Sunnybrook Health Sciences Center, University of Toronto, 2075 Bayview Ave., Room Rm AG 46, Toronto, M4N 3 M5 ON Canada; 2 Department of Surgery, Sunnybrook Health Sciences Center, University of Toronto, Toronto, ON Canada; 3 PanCuRx Translational Research Initiative, Ontario Institute for Cancer Research, Toronto, ON Canada; 4 Lunenfeld-Tanenbaum Research Institute, Mount Sinai Hospital, Toronto, ON Canada; 5 Hepatobiliary/pancreatic Surgical Oncology Program, University Health Network, Toronto, ON Canada; 6 Mechanical Engineering Department, Australian College of Kuwait, Kuwait City, Kuwait

**Keywords:** Texture Features, Pancreatic Ductal Adenocarcinoma, Overall Survival Prediction, Dissimilarity, Inverse Difference Normalized

## Abstract

**Background:**

To assess whether CT-derived texture features predict survival in patients undergoing resection for pancreatic ductal adenocarcinoma (PDAC).

**Methods:**

Thirty patients with pre-operative CT from 2007 to 2012 for PDAC were included. Tumor size and five texture features namely uniformity, entropy, dissimilarity, correlation, and inverse difference normalized were calculated. Mann–Whitney rank sum test was used to compare tumor with normal pancreas. Receiver operating characteristics (ROC) analysis, Cox regression and Kaplan-Meier tests were used to assess association of texture features with overall survival (OS).

**Results:**

Uniformity (*p* < 0.001), entropy (*p* = 0.009), correlation (*p* < 0.001), and mean intensity (*p* < 0.001) were significantly different in tumor regions compared to normal pancreas. Tumor dissimilarity (*p* = 0.045) and inverse difference normalized (*p* = 0.046) were associated with OS whereas tumor intensity (*p* = 0.366), tumor size (*p* = 0.611) and other textural features including uniformity (*p* = 0.334), entropy (*p* = 0.330) and correlation (*p* = 0.068) were not associated with OS.

**Conclusion:**

CT-derived PDAC texture features of dissimilarity and inverse difference normalized are promising prognostic imaging biomarkers of OS for patients undergoing curative intent surgical resection.

## Background

Cancers are phenotypically heterogeneous and their pattern of spatial heterogeneity varies with time [1]. Tumor heterogeneity is thought to be a key factor in the development of therapeutic resistance [2]. Tumor genomics from needle biopsy may be reflective of only a portion of the cancer’s characteristics. However, imaging has the distinct advantage of being non-invasive and providing an overview of the entire tumor. Therefore, imaging has increasingly been used to capture spatial heterogeneity of tumors [3]. The in-depth feature analysis of tumor sites has been brought into the “omics” terminology, called *radiomics*, which is defined as the high-throughput extraction of image features from radiographic images [1, 4, 5]. Imaging features can be derived from standard of care modalities such as contrast-enhanced computed tomography (CT), magnetic resonance imaging (MRI) and positron emission tomography (PET) without modification of the acquisition protocols making them less cost prohibitive [6–8]. For example, texture features from grey level co-occurrence matrices (GLCM) [9], which generate second-order statistical features have been used and improved [10] to quantify spatial texture of objects. There is an abundance of literature suggesting GLCM and other texture traits [11] are significantly associated with overall survival in lung [12], breast [13] and hepatic [14] carcinomas. However, to our best knowledge, there is paucity of studies to date about the potential prognostic value of CT texture features in pancreatic ductal adenocarcinoma (PDAC) [15].

PDAC has the lowest 5-year overall survival (OS) rate of any epithelial carcinoma at 7.7% [16] and surgical resection, applicable to < 30% cases [17], is the only potential cure [18] increasing OS to about 15–20% for resected cases [19]. More recently, neoadjuvant therapy has been introduced with the hope of extending survival for patients with resectable disease, allowing resection in patients with initially unresectable disease and selecting patients with different natural histories and chemosensitivities [20]. Since contrast-enhanced CT imaging is routinely used [21] for assessing resectability, staging and assessment of disease progression [22], a CT-derived quantitative imaging biomarker of OS could potentially provide a window into prognosis of PDAC.

The purpose of this study was to assess whether radiomic features from pre-operative contrast-enhanced CT in resectable PDAC patients were associated with overall survival. We hypothesized that pre-selected texture features are associated with the OS in resectable PDAC patients.

## Methods

### Patients

This retrospective study was approved by Sunnybrook Health Sciences Centre Research Ethics Board (reference number 400–2015). Written informed consent was waived.

Patients were identified from a database of all pancreatic resections performed at our institution. We included 30 consecutive patients who underwent curative intent surgical resection during 2007–2012, had pre-operative contrast-enhanced CT available for analysis (on average, one month prior to surgery). Cases of PDAC associated with an intraductal papillary mucinous neoplasm where excluded from this analysis. Also, patients who died within 3 months after surgery were excluded as the outcome may be significantly influenced by post-operative complications. Out of 30 patients, only 3 had gone under neoadjuvant therapy.

### Image acquisition

Patients underwent contrast-enhanced CT with a biphasic pancreas protocol. Positive oral contrast was given to patients starting 1 h before the scan time followed by 500 cc of water prior to scan. Pancreatic cancer boundaries were most consistently seen in the portal venous phase of acquisition so this was selected for region of interest (ROI) selection in this cohort. Intravenous contrast (Iohexol) (100–120 cc) at a rate of 4.0–5.0 cc per second was administered with automatic power injection. Scan resolution for the biphasic protocol was as follows; Pancreatic phase: helical 0.625 mm × 0.625 mm through pancreas, manual bolus tracking scanning triggered at 150 Hounsfield unit (HU) threshold; Portal phase: helical 0.625 mm × 0.625 mm through liver with 70 s delay; pitch was 0.984:1. CT images were reconstructed with 5 mm interval. Detector width was 40 mm and kV was 140 kVp for Pancreatic phase and 120 kVp for Portal phase. Examination was performed on a 64 row multidetector helical CT (GE Medical Systems, LightSpeed VCT, GE Healthcare).

### Image analysis

**Fig. 1:**
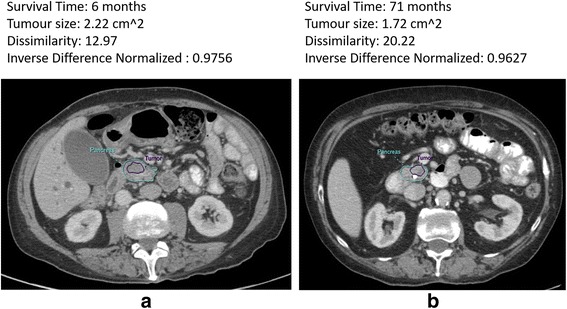
Representative patients contoured for tumor (purple line) and pancreas gland (cyan line) with specific survival and textural features shown on top of each panel. Both patients underwent a whipple procedure with vascular resection. **a** Patient with low survival time (6 months). **b** Patient with relatively high survival time (71 months)

For each primary cancer site, a ROI was drawn on all the slices with a visible tumor on the portal venous phase using an in-house developed contouring tool (ProCanVAS) [23]. ROIs were reviewed by a radiologist blinded to patient outcome. A ROI was also drawn to encompass normal pancreas on all slices that included tumor and 3 slices above and/or below the tumor depending on the location of tumor. In all cases, some hypointensity or relative contrast difference existed between background pancreas and the tumor. In cases where tumor boundary was not clear, boundary definition was facilitated by the presences of pancreatic or common bile duct cut-off and review of pancreatic phase images. A typical example of the contouring for two sample cases are presented in Fig. 1.

Tumor size plus five GLCM texture features [24] were preselected and calculated using an in house Matlab script (Mathworks Inc., USA, version 8.5.0.197613 - R2015a). GLCM feature set is one of the best known tools for texture analysis [25]. To calculate GLCM features, we used the bounding box around the ROIs annotated by the radiologist as the kernel, excluding the pixels in the bounding box located outside the ROI. The GLCM offset was set to be 1 pixel for the spatial relationship between adjacent pixels. Preselected features included entropy, dissimilarity, uniformity, correlation, and inverse difference normalized; selected based on previous literature suggesting high prognostic value in lung, colorectal, and prostate cancers [26–32]. In brief, these texture features provide a second order method for representing the conditional joint probabilities of all combination of grey levels. In brief, the probability measure can be defined as:

$$ \Pr (x) = \left\{\left( Cij\left|\right(\delta, \theta \right)\right\} $$

Where δ and θ are interpixel distance and orientation, respectively. Cij, the co-occurrence probability between grey levels i and j, is defined as:

$$ {C}_{i j} = \frac{P_{i j}}{{\displaystyle {\sum}_{i, j=1}^G}{P}_{i j}} $$

Pij is the number of occurrences of grey levels i and j within the given window, given a certain (δ,θ) pair, and G is the quantized number of grey levels. The sum in the denominator thus represents the total number of grey level pairs (i,j) within the window. Statistics were applied to the co-occurrence matrix following [24] as presented in Table 1. Voxels with HU < −10 and > 500 were filtered from analysis in all cases to remove the fluid and stents placed before the preoperative CT [33]. Excluding voxels with HU < −10 is crucial because fat both surrounds and interdigitates between lobules of pancreatic tissue. This will produce significant texture effect in pancreatic tissue based on a process independent of cancer and more related to fatty infiltration which can be quite variable based on age and metabolic status of the patient. When contouring the pancreas, the radiologists can find it difficult to be so precise as to eliminate every voxel of fat at the margin thus small changes in contour at a fat boundary where contrast enhanced tissue and non-contrast enhanced fat will have large variations in HU could produce erroneous texture measurements. We based the choice of -10HU on published thresholds for intralesional fat detection for angiomyolipoma which is a fat containing renal tumor [34]. Values of features from the largest cross section of the tumor were calculated across slices in which each ROI appeared. These values were used for the statistical analysis.

**Table 1:** Grey level co-occurrence texture features. All summations are over all (i,j) pairs

Parameter	Mathematical definition
Uniformity	${\displaystyle \sum_{i, j=1}^G}{C_{i j}}^2$
Entropy	${\displaystyle \sum_{i, j=1}^G}{C}_{i j}\ log{C}_{i j}$
Dissimilarity	${\displaystyle \sum_{i, j=1}^G}{C}_{i j}\ \left| i- j\right|$
Inverse Difference Normalized	${\displaystyle \sum_{i, j=1}^G}\frac{C_{i j}}{1+\left| i- j\right|{}^2/{G}^2}$
Correlation	${\displaystyle \sum_{i, j=1}^G}\frac{\left( i-\mu x\right)\left( j-\mu y\right){C}_{i j}}{\sigma x\sigma y}$

### Statistical analysis

The texture features in tumor and normal pancreas were compared using a Mann–Whitney rank test. A Wald test with Cox regression model was used to test for associations between each texture feature and survival. A two-sided *p*-value of less than 0.05 was considered statistically significant. Receiver operating characteristics (ROC), including area under the curve (AUC), was used to study the prognostic value of each texture parameter. The medians were used for Kaplan-Meier plots. Data management and statistical analysis were conducted using IBM SPSS Statistics package (version 23, SPSS Inc., Chicago, IL, USA).

## Results

The demographic information of the cohort is shown in Table 2. Tumor region and the normal pancreas were used for analysis. The median (interquartile range) of HU was 71 (61–82) and 57 (41–63) in normal pancreas and tumor, respectively (*p* < 0.001). The HU was significantly higher in normal tissue than tumor regions in all patients (*p* < 0.001) for the portal venous phase. Tumor was significantly different than normal pancreas, as shown in Table 3; uniformity (*p* < 0.001), entropy (*p* = 0.009), and correlation (*p* < 0.001). However, the difference in dissimilarity (*p* = 0.530) and inverse difference normalized (*p* = 0.511) were not significant.

**Table 2:** Demographic information of studied cohort

Age (years)	Mean ± Standard deviation	69 ± 8
Sex	Female/Male/Total	13/17/30
Vascular resection	Yes/No/Total	15/15/30
Size (cm2)	Mean ± Standard Deviation	2.13 ± 1.88
Grade	G1/G2/G3/Total	3/19/8/30
Nodes Sampled (Per Patient)	Mean ± Standard Deviation	25 ± 11
Patients with Negative/Positive Nodes	N0/N1	6/24
Margin	R2/R1/R0	0/16/14
Survival Time (months)	Mean ± Standard Deviation	31 ± 25

Nodes Sampled is the number of nodes taken from each patient. Patients with Negative Nodes is the number of patients whose sampled nodes were all negative. Patients with Positive Nodes is the number of patients who had at least one positive sampled node

**Table 3:** Comparison of normal and tumor tissues (Entries in bold were significant)

Texture feature	Tumor tissue median (interquartile range)	Normal tissue median (interquartile range)	Tumor vs Normal comparison *p*-value (Rank sum test)
Uniformity	0.181 (0.165–0.192)	0.210 (0.189–0.225)	**<0.001**
Entropy	{−0.758 (−0.987-0.681)} × 10^−3^	{−0.611 (−0.746-0.508)} × 10^−3^	**0.009**
Dissimilarity	0.286 (0.249–0.311)	0.270 (0.223–0.304)	0.530
Correlation	0.393 (0.267–0.464)	0.486 (0.430–0.591)	**<0.001**
Inverse Difference Normalized	0.859 (0.845–0.877)	0.866 (0.849–0.889)	0.511
Mean Intensity	55.988 (41.099–62.617)	70.255 (60.452–81.506)	**<0.001**

**Fig. 2:**
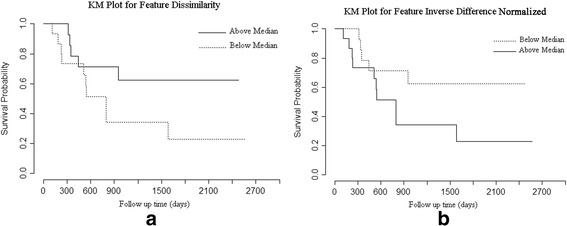
Kaplan-Meier plot of cumulative survival for **a**) dissimilarity and **b**) inverse difference normalized

Wald-test in Cox regression analysis on tumor texture parameters showed dissimilarity (coefficient = −0.1292, *p*-value = 0.045) and inverse difference normalized (coefficient = 71.81, *p*-value = 0.046) were significantly associated with OS as shown in Table 4. Kaplan-Meier plots of cumulative survival for significant tumour features are provided in Fig. 2. Also, size of the tumor (coefficient = 0.000627, *p*-value = 0.611) and the average intensity of the tumor (coefficient = −0.011, *p*-value = 0.366) were not significantly associated with OS. Other tumor texture features were not significantly associated with survival with the coefficients and *p*-values as follows: uniformity (coefficient = −105.5, *p*-value = 0.334), entropy (coefficient = 0.324, *p*-value = 0.330), correlation (coefficient = 4.013, *p*-value = 0.068).

**Table 4:** Cox regression for survival analysis using texture features and size of tumor **(**Entries in bold were significant)

Parameter	B value	Standard error	Wald	*p*-value
Uniformity	−105.5	109.2	0.93	0.334
Entropy	0.3240	0.333	0.95	0.330
Dissimilarity	**−0.1292**	**0.065**	**4.01**	**0.045**
Correlation	4.013	2.195	3.34	0.068
Inverse Difference Normalized	**71.81**	**36.04**	**3.97**	**0.046**
Tumor Size	0.000627	0.00123	0.26	0.611
Tumor Intensity	−0.011	0.013	0.82	0.366

Among the studied features, dissimilarity (AUC = 0.716) and inverse difference normalized (AUC = 0.716) showed maximal predictive value for predicting OS. AUC for uniformity, entropy, and correlation were 0.560, 0.569, and 0.680, respectively. Table 5 represents details of the ROC analysis.

**Table 5:** Receiver operating characteristic analysis of texture features and size for predicting survival outcome **(**Entries in bold were found significant)

Parameter	Sensitivity	Specificity	AUC	Threshold	95% CI	*p*-value
Uniformity	0.6	0.4	0.560	0.002	0.230–0.650	0.576
Entropy	0.6	0.533	0.569	5.901	0.360–0.778	0.520
Dissimilarity	**0.667**	**0.733**	**0.716**	**16.311**	**0.528–0.903**	**0.044**
Correlation	0.533	0.733	0.68	0.610	0.484–0.875	0.093
Inverse Difference Normalized	**0.667**	**0.733**	**0.716**	**0.969**	**0.528–0.903**	**0.044**
Tumor Size	0.533	0.533	0.538	154.761	0.326–0.750	0.724
Tumor intensity	0.533	0.533	0.524	58.462	0.313–0.736	0.820

**Fig. 3:**
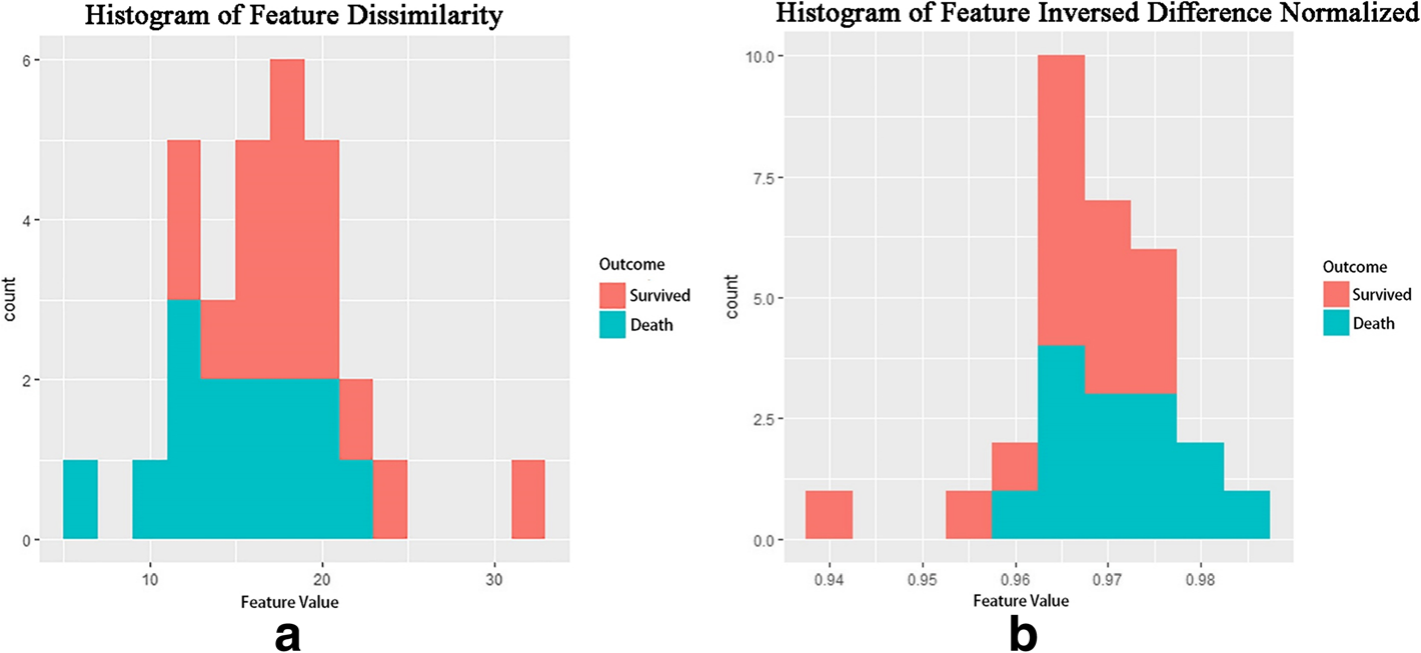
Histograms of significant features **a**) dissimilarity and **b**) inverse difference normalized. The figure also illustrates the distribution of survival across the features values

Figure 3 shows the histogram of two significant features namely dissimilarity and inverse difference normalized. This figure also illustrates the distribution of survival across the features values.

## Discussion

In our study, we used GLCM textural analysis from venous phase contrast-enhanced CT and found that dissimilarity and inverse difference normalized were associated with OS in a cohort of resectable PDAC patients. These features provided a stronger association with OS than tumor intensity and tumor size.

We found that less inverse difference normalized and greater dissimilarity are associated with longer OS. As texture analysis has not been used in the context of resectable PDAC, there were no studies to compare our findings. However, we can compare our results with other studies that followed a similar hypothesis in other types of cancer. Our findings are consistent with previous radiomic studies for lung, breast, and other cancer site. Different studies have also shown that dissimilarity feature extracted from ROIs in PET/CT has a positive correlation with survival time for non-small cell lung cancer [35, 36] and Multi–Cancer site patient cohorts [37]. It is important to note that the underlying meaning of imaging texture cannot be reduced to a mean regional intensity but is estimated from the probability of the occurrence of a specific pattern of intensities which can explain why mean intensity was not associated with survival outcome in our study and others. Such texture features may not be readily visible on standard grey scale images.

Given the challenging management of PDAC which presents late and is highly lethal, it is hoped that gathering clues of factors associated with overall survival from CT could augment the ability for treatment decision making [38] and aid in prognosticating treatment scenarios. Our findings show that with minimal cost and with no additional imaging burden, textural feature analysis of routine contrast-enhanced CT imaging before surgery may provide useful information for PDAC patients undergoing curative intent surgical resection. Our results show that textural analysis is more strongly associated with OS than tumor size. Such informed decision may help in identifying therapeutic plan for patients, for example, considering targeted adjuvant or neoadjuvant treatments in some patients with predicted poor prognosis. On the other hand, it may help in identifying patients with very poor prognosis who are unlikely to benefit from surgery; in these patients chemotherapy or radiation may be the optimal treatment modality [39]. Certainly for clinical application in personalized medicine, a wider repertoire of treatment options with better survival is a fundamental challenge in this nearly uniformly lethal disease. As our understanding of imaging biomarkers continues to unfold, it is hoped that this may provide more insight into the likely benefit of new therapeutic regimes in subpopulations of patients.

This study has limitations. The small sample size limits our ability to perform a multivariate analysis and thus additional stratification based on tumor extent such as resectable versus borderline resectable stratification could not be evaluated. Despite a relatively small sample size, we found strong association of the textural features with OS. The findings of this study encourage investigating the association of a wider range of radiomic features with survival and intermediate factors such as radiogenomics in a larger sample size. Also the reported findings should be verified independently in future studies. Further work is also needed to address the repeatability of these quantitative imaging biomarkers as part of a biomarker validation process [40]. There is always a risk of achieving statistically positive results by chance with small sample sizes. We have tried to restrict the number of features being evaluated by choosing features that have already been shown to be prognostic in a variety of other adenocarcinomas [12, 26]. The fact that some of the texture features associated with OS match those in other adenocarcinomas is encouraging. Finally, there is a lack of understanding on the underlying relationship of texture features and histology, genomics and proteomics of PDAC which requires further work.

## Conclusions

CT-derived PDAC texture features of dissimilarity and inverse difference normalized are promising prognostic imaging biomarkers of OS for patients undergoing curative intent surgical resection.

## Abbreviations

AUC: Area under the curve
CT: Computed tomography
GLCM: Grey-level co-occurrence matrix
HU: Hounsfield unit
MRI: Magnetic resonance imaging
OS: Overall survival
PDAC: Pancreatic ductal adenocarcinoma
PET: Positron emission tomography
ROC: Receiver operating characteristics
ROI: Region of interest
